# Genome-wide identification and expression analysis of *BrAGC* genes in *Brassica rapa* reveal their potential roles in sexual reproduction and abiotic stress tolerance

**DOI:** 10.3389/fgene.2022.1044853

**Published:** 2022-10-26

**Authors:** Xiaoyu Wu, Lianhui Pan, Xinping Guo, Ting Li, Jiali Li, Qiaohong Duan, Jiabao Huang

**Affiliations:** ^1^ State Key Laboratory of Crop Biology, Shandong Agricultural University, Tai’an, Shandong, China; ^2^ College of Horticulture Science and Engineering, Shandong Agricultural University, Tai’an, Shandong, China

**Keywords:** *Brassica rapa*, AGC protein kinase, gene family, bioinformatics, expression profile

## Abstract

AGC protein kinases play important roles in regulating plant growth, immunity, and cell death. However, the function of AGC in *Brassica rapa* has not yet been clarified. In this study, 62 *BrAGC* genes were identified, and these genes were distributed on 10 chromosomes and divided into six subfamilies. Analysis of gene structure and conserved motifs showed that the activation segment of *BrAGC* genes was highly conserved, and genes of the same subfamily showed higher sequence and structural similarity. Collinearity analysis revealed that *BrAGCs* were more closely related to *AtAGCs* than to *OsAGCs*. Expression profile analysis revealed that *BrAGCs* were preferentially expressed in flowers and *BrAGC26*, *BrAGC33*, and *BrAGC04* were preferentially expressed in the stigma; the expression of these genes was significantly upregulated after self-incompatibility pollination, and the expression of *BrAGC13* and *BrAGC32* was significantly upregulated after cross-pollination. In addition, several typical *cis*-elements involved in the stress response were identified in *BrAGC* promoters. The expression levels of *BrAGC37* and *BrAGC44* significantly varied under different types of abiotic stress. Collectively, we identified that *BrAGC26*, *BrAGC33,* and *BrAGC44* have the greatest potential in regulating pollen-pistil interaction and abiotic stress tolerance, respectively. Our findings will aid future functional investigations of *BrAGCs* in *B. rapa*.

## Introduction

Protein phosphorylation is a ubiquitous and significant post-translational modification that plays an important role in intracellular signal transduction. The activity of protein kinases is turned on or off by phosphorylation and dephosphorylation, and this mediates the responses to the internal and external environment. AGC protein kinases, which were described for the first time by Steven Hanks and Tony Hunter (Hanks and Hunter, 1995), are an important kinase family and have attracted much research attention. The activation of most AGC kinases involves the phosphorylation of two highly conserved motifs, the activation segment (T-loop) in the catalytic domain, and the hydrophobic motif located behind the catalytic domain (Pearce et al., 2010). In mammals and yeast, AGC protein kinase is an important downstream effector that senses intracellular second messengers such as cAMP, cGMP, phospholipids, and Ca^2+^, and it is activated by second messengers (Bögre et al., 2003). In plants, AGC protein kinases have been shown to be involved in regulating plant growth, immunity, and cell death. In 2003, 39 AGC protein kinases in *Arabidopsis thaliana* (*AtAGC*) were identified for the first time, and these were divided into six subfamilies: AGCVI, AGCVII, AGCVIIIa, AGCVIIIb, other AGCs, and PDK1 (Bögre et al., 2003).

Previous studies have shown that plant AGC protein kinases play an important role in responses to various environmental stresses and in regulating plant immunity. Concentrations of reactive oxygen species (ROS) increase when plants are exposed to stress. Oxidative signal-inducible kinase (OXI), a member of the AGC protein kinase family, plays a role in ROS-initiated downstream signaling when plants are exposed to stress. In *A. thaliana*, *AtOXI1* mediates the transduction of oxidative stress signals under abiotic stress or during pathogen invasion as well as responses to stress (Rentel et al., 2004; Petersen et al., 2009). When plants are exposed to high-light stress, OXI can induce programmed cell death (PCD) to prevent damage induced by high light (Shumbe et al., 2016). In rice, ROS and chitin elicitors can induce the transient phosphorylation of *OsOxi1*, which plays a positive role in regulating plant disease resistance (Matsui et al., 2010). In tomato, AvrPto-dependent Pto-interacting protein 3 (Adi3) is a negative regulator of PCD caused by *Pseudomonas syringae pv. Tomato* (*Pst*), and it plays a role in regulating plant immunity (Devarenne et al., 2006). In wheat, *TaAGC1* plays a positive regulatory role in host resistance by regulating the expression of ROS and resistance-related genes (Zhu et al., 2015). Previous studies have also shown that AGCs play a role in plant reproduction. In *A. thaliana*, AGC1.5 and AGC1.7 regulate the ROP/Rac small GTPase (ROP) signaling pathway through phosphorylation positive feedback to regulate the apical growth of pollen tubes (Zhang et al., 2009; Li et al., 2018). In addition, AGC protein kinases also regulate the process of plant growth and development by regulating auxin polar transport (Friml et al., 2004), sensing blue light signals, mediating plant phototropism, regulating the distribution of chloroplasts and stomatal opening and closing (Sakai et al., 2001; Inada et al., 2004), and modifying cell proliferation and embryonic development to regulate seed size (Zhang et al., 2020). The published plant AGCs are listed in Supplementary Table S1. These studies have shown that AGCs play multiple roles and regulate divergent biological processes in plants.

Chinese cabbage (*Brassica rapa*) is widely grown in Asia and is exposed to various types of stress during its growth. However, no studies to date have examined AGC protein kinases in Chinese cabbage. In this study, *BrAGC* genes were identified at the whole-genome level, and the physicochemical properties, structure of genes and proteins, and expression profiles were analyzed. The results of this study provide basic information that will aid future studies of BrAGC protein kinases.

## Materials and methods

### Identification of *AGC* gene family members in *B. rapa*

Whole-genome sequences, gff3 genome annotation data, and AGC amino acid (aa) sequences of *B. rapa*, *A. thaliana*, and *Oryza sativa* were downloaded from EnsemblPlants (http://plants.ensembl.org/). *BrAGC* genes were screened from the *B. rapa* genome using the BLASTP program with *A. thaliana AGC* (*AtAGC*) aa sequences as input to predict *B. rapa AGC* (*BrAGC*) genes. Pfam (http://pfam.xfam.org/) (Mistry et al., 2021) was used to predict the structure of the proteins of the predicted genes; candidate genes were screened based on the conserved domains, and *BrAGC* genes were determined. The physicochemical properties of *BrAGC* genes, including their isoelectric points (pI) and relative molecular masses, were analyzed using the ExPASy website (http://www.expasy.org/) (Artimo et al., 2012).

### Phylogenetic tree construction, conserved motif, gene structure, and *cis*-element analysis

The aa sequences of *BrAGC*s, *AtAGC*s, and *OsAGC*s were aligned using the MUSCLE algorithm in MEGA X (Kumar et al., 2018), and an un-rooted phylogenetic tree was constructed using the maximum likelihood method in MEGA X with 1,000 bootstrap replicates. The aa sequences of the *BrAGC* genes were uploaded to the MEME online website (http://meme-suite.org/) (Bailey and Elkan, 1994), and the number of motifs was set to 10 to analyze the conserved motifs of *BrAGC*. The upstream 2000-bp sequence of each *BrAGC* gene was analyzed using the online PlantCARE database (http://bioinformatics.psb.ugent.be/webtools/plantcare/html/) (Rombauts et al., 1999) with default parameters. *BrAGC* gene structure and conserved motifs were visualized using TBtools (Chen et al., 2020).

### Chromosomal localization, gene replication, and collinearity analysis

The location of the *BrAGC* gene on each chromosome was extracted from the *B. rapa* gff3 genome annotation information using TBtools (Chen et al., 2020). The MCScanX (Multiple Collinearity Scan toolkit) plug-in in TBtools was used to analyze the relationships among duplicate genes within *B. rapa* and inter-species collinearity, and the Circos plug-in was used to visualize the results of the analysis. Non-synonymous substitutions (Ka) and synonymous substitutions (Ks) values were calculated for duplicated gene pairs using TBtools. Gene selection pressure analysis was performed using the Ka/Ks ratio. If Ka/Ks > 1, the gene has experienced positive selection; if Ka/Ks = 1, the gene has undergone neutral evolution; and if Ka/Ks < 1, the gene has experienced purifying selection (Hurst, 2002).

### Protein–protein interaction networks and gene ontology analysis

STRING online website (http://cn.string-db.org) was used to predict protein–protein interaction (PPI) relationships with default parameters, and Cytoscape v3.9.1 was used to construct the interaction network (Shannon et al., 2003). Gene Ontology (GO) analysis of *BrAGC* genes with default parameters was conducted using the online website DAVID (http://david.ncifcrf.gov) (Dennis et al., 2003).

### Plant material and stress treatments

The first-generation hybrid cultivar “848” of *B. rapa* with stable self-incompatibility was used for stress treatments. The plump seeds were sown in nursery pots filled with substrate and cultivated in a plant incubator (16-h light/8-h dark photoperiod at 25°C, light intensity 2000 lx), When the seedlings had six leaves, seedlings with similar growth status were subjected to stress treatments. The seedlings were placed in a hydroponic system with 150 mM NaCl to simulate salt stress and 15% PEG 6000 to simulate drought conditions. For the cold stress treatment, plants were exposed to 4°C. The duration of all stress treatments was 2, 4, 6, and 12 h. The stigma of ZY15 was pollinated using the pollen of ZY15 and 14CR, which corresponded to self-incompatibility (SI) and cross-pollination (CP), respectively. There were three biological replicates for each treatment group, and samples were stored at −80°C for subsequent RNA extraction.

### Total RNA extraction and qRT-PCR

Total RNA was extracted using the SteadyPure Plant RNA Extraction Kit (Code No. AG21017, Accurate Biotechnology, China); samples were reverse-transcribed using TransScript^®^ Uni All-in-One First-Strand cDNA Synthesis SuperMix for qPCR (TransGen, AU341-02, Beijing, China) for qRT-PCR analysis. qRT-PCR was performed on a qTOWER3 qPCR machine (Analytikjena, Germany) using the ChamQ SYBR qPCR Master Mix (Q711-03, Vazyme, China), and *BrActin2* was used as the reference gene, analysis of the relative expression levels of each gene was conducted using the 2^−ΔΔ^Ct method. The specific primer sequences are listed in Supplementary Table S2.

### Expression analysis

The transcriptome sequences of *B. rapa* of different tissues from NCBI GEO (https://www.ncbi.nlm.nih.gov/geo/) (Barrett and Edgar, 2006) with the accession number GSE43245 (Tong et al., 2013) were downloaded, and the data were normalized using the transcripts per million (TPM) method. The unpollinated stigmas and stigmas at 5, 10, and 20 min after SI and CP of *B. rapa* were collected and stored in liquid nitrogen. Three biological replicates were collected for each sample. The samples were subjected to transcriptome sequencing on the Illumina NovaSeq 6000 platform (unpublished) by BioMarker Technologies (Beijing, China). The number of mapped reads and transcript lengths in the samples were normalized after sequencing. The abundance of transcripts was measured using fragments per kilobase of transcript per million fragments mapped (Trapnell et al., 2010). Gene expression profile heatmaps were built using TBtools (Chen et al., 2020).

## Result

### Genome-wide identification and physicochemical characterization of *B. rapa AGC* genes

A total of 62 *BrAGC* genes were screened from the *B. rapa* genome using the BLAST program with the *AtAGC* gene sequences as input. The genes were renamed as *BrAGC01*–*BrAGC62* and divided into six subfamilies. The physicochemical properties of *AtAGC* genes and *BrAGC* genes were analyzed and listed in Table 1; the lengths of the *BrAGC* proteins ranged from 76 aa (*BrAGC59* and *BrAGC61*) to 1281 aa (*BrAGC27*), the molecular weight ranged from 8.30 kDa (*BrAGC59*) to 141.65 kDa (*BrAGC27*), and the isoelectric point (pI) ranged from 4.85 (*BrAGC61*) to 9.52 (*BrAGC28* and *BrAGC62*).

**TABLE 1:** Basic information of *AGC* genes identified in *B. rapa* and *A. thaliana*.

*B. rapa* AGC genes									*A. thaliana* homologous AGC genes							
Subfamily	Gene ID	Gene name	Chromosome (Chr)	Start	End	pI	Amino acid length (aa)	Molecular weight (average)	Gene ID	Gene name	Chr	Start	End	pI	Amino acid length (aa)	Molecular weight (average)
AGCVI	Bra040057	BrAGC09	A01	27741102	27742801	6.21	460	51,920.51	At3g08730	S6K-1	3	2651321	2654212	5.81	465	52,587.99
	Bra040056	BrAGC10	A01	27744350	27746085	6.48	407	45,568.41								
	Bra029713	BrAGC31	A05	22551724	22553415	5.89	461	52,161.58								
	Bra029712	BrAGC32	A05	22554798	22556631	6.48	452	50,708.98	At3g08720	S6K-2	3	2648372	2651231	6.27	471	53,037.42
AGCVII	Bra033489	BrAGC03	A01	11651412	11667121	8.79	1011	116,298	At4g14350	NDR-1	4	8256021	8260927	8.54	551	63,180.35
	Bra010763	BrAGC48	A08	16298200	16300948	8.3	550	63,155.36								
	Bra015279	BrAGC53	A10	2736640	2741316	6.91	567	65,140.73	At1g03920	NDR-2	1	1001116	1004410	6.1	569	65,029.43
	Bra033404	BrAGC54	A10	3842866	3847244	6.15	288	33,010.24								
	Bra014937	BrAGC45	A07	5077313	5079952	8.65	551	63,404.35	At3g23310	NDR-3	3	8338871	8343737	8.78	568	65,322.56
	Bra023736	BrAGC05	A01	19467439	19468734	8.59	207	24,078.78								
	Bra038998	BrAGC43	A07	940,004	942,811	5.53	528	60,720.69	At2g19400	NDR-4	2	8399100	8402664	5.76	527	60,658.71
	Bra036723	BrAGC49	A09	5415304	5417949	5.97	475	54,924.42								
	Bra036473	BrAGC42	A07	170,364	179,142	6.19	1205	138,582.5	At2g20470	NDR-5	2	8826017	8829771	5.73	569	64,843.13
	Bra031123	BrAGC52	A09	32372044	32374754	5.8	540	61,444.51								
	Bra011411	BrAGC01	A01	2281829	2286066	5.53	519	60,097.66	At4g33080	NDR-6	4	15959906	15964819	5.52	519	60,431.09
	Bra032411	BrAGC50	A09	21646890	21650306	9.15	562	64,819.16	At1g30640	NDR-7	1	10860619	10865074	9.15	562	64,911.29
	Bra031468	BrAGC04	A01	17180264	17183236	5.56	523	59,978.47	At5g09890	NDR-8	5	3085544	3089119	5.62	515	59,252.55
	Bra040471	BrAGC60	Scaffold000209	57,645	60,083	4.88	408	46,676.84								
AGC VIIIa	Bra035592	BrAGC13	A02	7387360	7388958	8.49	362	40,554.58	At5g55910	AGC1-1	5	22639813	22642414	8.47	498	54,816.38
	Bra002868	BrAGC55	A10	7051656	7053232	8.58	500	55,110.6								
	Bra028955	BrAGC16	A03	5585868	5587954	8.56	492	54,127.66								
	Bra026417	BrAGC02	A01	9419043	9420659	8.51	513	56,614.35	At4g26610	AGC1-2	4	13424477	13427402	8.55	506	55,946.54
	Bra024918	BrAGC41	A06	24247150	24249048	8.7	554	61,383.83	At5g47750	AtPK5	5	19339703	19342410	8.96	586	64,556.69
	Bra025288	BrAGC40	A06	22372952	22381747	8.97	588	65,559.88	At3g27580	AtPK7	3	10217417	10220061	8.72	578	64,286.29
	Bra037631	BrAGC20	A04	18412280	18414936	6.31	648	71,779.75	At2g44830	AGC1-3	2	18489607	18493163	6.29	765	84,706.11
	Bra004872	BrAGC21	A05	2228412	2231516	6.06	701	77,646.7								
	Bra025621	BrAGC17	A04	7449137	7451050	7.56	493	54,783.34	At5g40030	AGC1-4	5	16026047	16028483	8.55	499	55,495.47
	Bra026056	BrAGC36	A06	6310104	6311791	8.61	443	49,761.83	At1g16440	AGC1-6	1	5615808	5617672	8.68	499	56,129.7
	Bra008404	BrAGC14	A02	15389477	15391784	9.21	644	71,901.32	At1g79250	AGC1-7	1	29810134	29812683	8.43	555	61,068.96
	Bra038744	BrAGC07	A01	26339356	26341615	8.83	572	62,987.45	At3g12690	AGC1-5	3	4030387	4033387	9.08	577	63,767.51
	Bra034743	BrAGC29	A05	21240076	21242354	9.07	571	62,967.37								
	Bra040842	BrAGC61	Scaffold000277	8382	8609	4.85	75	8440.5	At2g36350	AGC1-9	2	15238093	15242195	8.14	949	104,521
	Bra039602	BrAGC59	Scaffold00017	276,079	276,306	4.88	75	8295.34								
	Bra033907	BrAGC25	A05	15090187	15090666	5.01	159	17,865.2								
	Bra005275	BrAGC22	A05	4491881	4492369	5.01	162	18,175.55								
	Bra006955	BrAGC51	A09	26473931	26478245	8.2	1044	115,873.3	At3g52890	KIPK	3	19607897	19612217	7.3	934	102,693.9
	Bra005280	BrAGC23	A05	4514491	4517689	8.44	888	97,973.73								
	Bra028538	BrAGC11	A02	963,034	965,794	7.73	895	98,968.03								
	Bra019641	BrAGC35	A06	5342000	5344547	8.12	788	87,050.76								
	Bra009522	BrAGC58	A10	17067972	17071022	7.55	896	99,839.21	At5g03640	AGC1-8	5	927,136	930,921	7.3	926	102,161.5
	Bra019427	BrAGC38	A06	13906141	13909715	6.54	441	49,048	At3g44610	AGC1-12	3	16188129	16192365	6.74	451	50,015.05
	Bra041025	BrAGC62	Scaffold000388	2670	4148	9.52	492	55,573.53	At1g53700	AtPK3	1	20048434	20050294	9.15	476	53,820.3
	Bra027344	BrAGC28	A05	20437266	20438705	9.52	479	54,213.58	At3g14370	AGC1-11	3	4797828	4799612	9.53	480	54,426.8
	Bra034312	BrAGC18	A04	11831526	11833909	9.01	529	59,841.89	At2g26700	AGC1-10	2	11368474	11371124	9.05	525	59,294.38
	Bra005403	BrAGC24	A05	5317911	5319529	9.45	439	49,364.5	At2g34650	PID	2	14589736	14592140	9.42	438	49,271.48
	Bra021925	BrAGC19	A04	15212235	15213831	9.09	439	49,187.29								
AGC VIIIb	Bra035740	BrAGC26	A05	16853185	16854396	9.11	403	45,183.48	At3g20830	AGC2-4	3	7284681	7286442	9.28	408	45,889.49
	Bra018898	BrAGC33	A06	1382085	1382948	8.5	287	32,915.61								
	Bra019474	BrAGC37	A06	13180501	13189507	7.88	963	107,813.1	At3g45780	PHOT1	3	16816721	16824210	7.54	996	111,688.8
	Bra015120	BrAGC44	A07	3332989	3334309	5.52	414	46,784.85	At3g25250	AGC2-1	3	9195334	9197126	5.66	421	47,559.73
	Bra006782	BrAGC15	A03	5031928	5036536	8.54	902	101,083	At5g58140	PHOT2	5	23523825	23533111	8.72	898	100,789
	Bra002669	BrAGC56	A10	8318839	8323864	6.52	882	98,564.92								
	Bra020388	BrAGC12	A02	6512671	6517209	6.42	881	98,821.24								
									At4g13000	AGC2-2	4	7598004	7599447	5.98	372	42,159.02
									At1g51170	AGC2-3	1	18953458	18955053	8.69	404	45,664.98
PDK 1	Bra009447	BrAGC57	A10	16680343	16682826	8.59	490	54,406.99	At5g04510	PDK1-1	5	1286843	1289908	6.4	305	34,244.14
	Bra029850	BrAGC30	A05	21990187	21992642	8.59	481	53,627.9	At3g10540	PDK1-2	3	3289677	3292909	8.12	486	54,440.82
	Bra034125	BrAGC08	A01	27325086	27327487	7.65	488	54,390.63								
AGC other	Bra010074	BrAGC39	A06	19183274	19188241	5.37	1125	125,307.6	At5g62310	IRE	5	25023155	25028686	5.36	1168	130,147.2
	Bra022251	BrAGC27	A05	18821697	18827796	5.69	1281	141,654.3	At3g17850	IRE-H1	3	6109409	6116529	5.54	1296	143,792.4
	Bra021278	BrAGC06	A01	22828567	22834571	5.46	1256	139,623.9								
	Bra018730	BrAGC34	A06	2280908	2286437	5.99	927	105,067.2	At1g48490	IRE-3	1	17921900	17928954	5.52	1235	137,348.6
	Bra014141	BrAGC46	A08	3150758	3156652	5.42	1213	134,970.5								
	Bra014042	BrAGC47	A08	4202957	4208136	5.66	1057	118,594.8	At1g45160	IRE-4	1	17083077	17090588	5.66	1067	120,311.8

### Chromosomal distribution, gene duplication, and phylogenetic analysis

**FIGURE 1:**
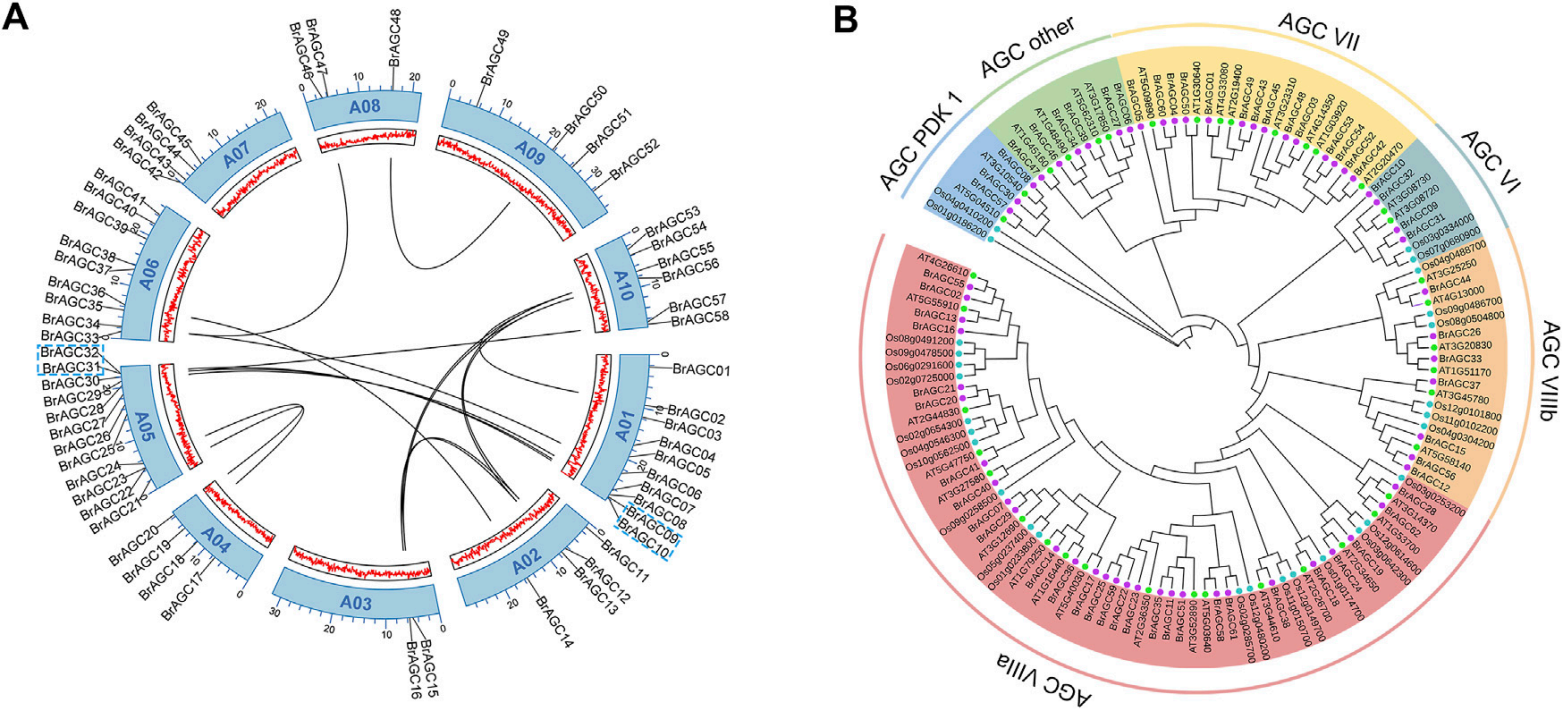
Chromosome localization and phylogenetic analysis of *BrAGC* genes **(A)** Gene distribution and duplication events of *BrAGC* genes. The gray line is the segmentally duplicated gene pair, and the tandemly duplicated gene is indicated by blue dotted lines. **(B)** Phylogenetic tree from the maximum likelihood analysis of the *AGC* gene family in *B. rapa*, *A. thaliana*, and *O. sativa*.

According to the gff3 genome annotations, 58 *BrAGC*s were mapped to the chromosomes of *B. rapa*, and the remaining four genes were mapped to the chromosome scaffold (Figure 1A). Among them, 12 *BrAGC*s were found on chromosome A05, accounting for 19% of all *BrAGC* genes. This was followed by A01 (10 genes); A06 (9 genes); A10 (6 genes); A02, A04, A07, and A09 (4 genes); A08 (3 genes); and A03 (2 genes). Gene duplication is considered a major cause of gene family expansion in the genome (Cannon et al., 2004). To clarify the associations among *BrAGCs*, we performed intraspecific collinearity analysis and detected two tandem duplications and 17 chromosome segmental duplications in *BrAGCs* (Figure 1A; Supplementary Table S3). The number of segmental duplications was 8.5 times the number of tandem duplications; this might be a major reason for the expansion of *BrAGC* genes in the *B. rapa* genome. We analyzed selection on gene pairs in *BrAGC* using the Ka/Ks ratio. Ka was much less than Ks (Ka/Ks < 1) for all tandem repeat gene pairs and segmentally duplicated gene pairs; this indicates that each replicated gene pair has experienced strong purifying selection; the Ka/Ks ratio was highest for *BrAGC34*/*BrAGC46* (0.61) (Supplementary Table S3).

To further clarify the evolutionary relationships among *BrAGC* genes, we performed phylogenetic analysis on the *AGC* genes in *B. rapa*, *A. thaliana*, and *O. sativa* and constructed an ML tree (Figure 1B). We divided *BrAGC* genes into six subfamilies: AGCVI, AGCVII, AGCVIIIa, AGCVIIIb, other AGCs, and AGCPDK1. The largest and smallest subfamilies were AGCVIIIa and AGCPDK1, which contained 28 and three genes, respectively.

### Gene structure and conserved motif analysis

**FIGURE 2:**
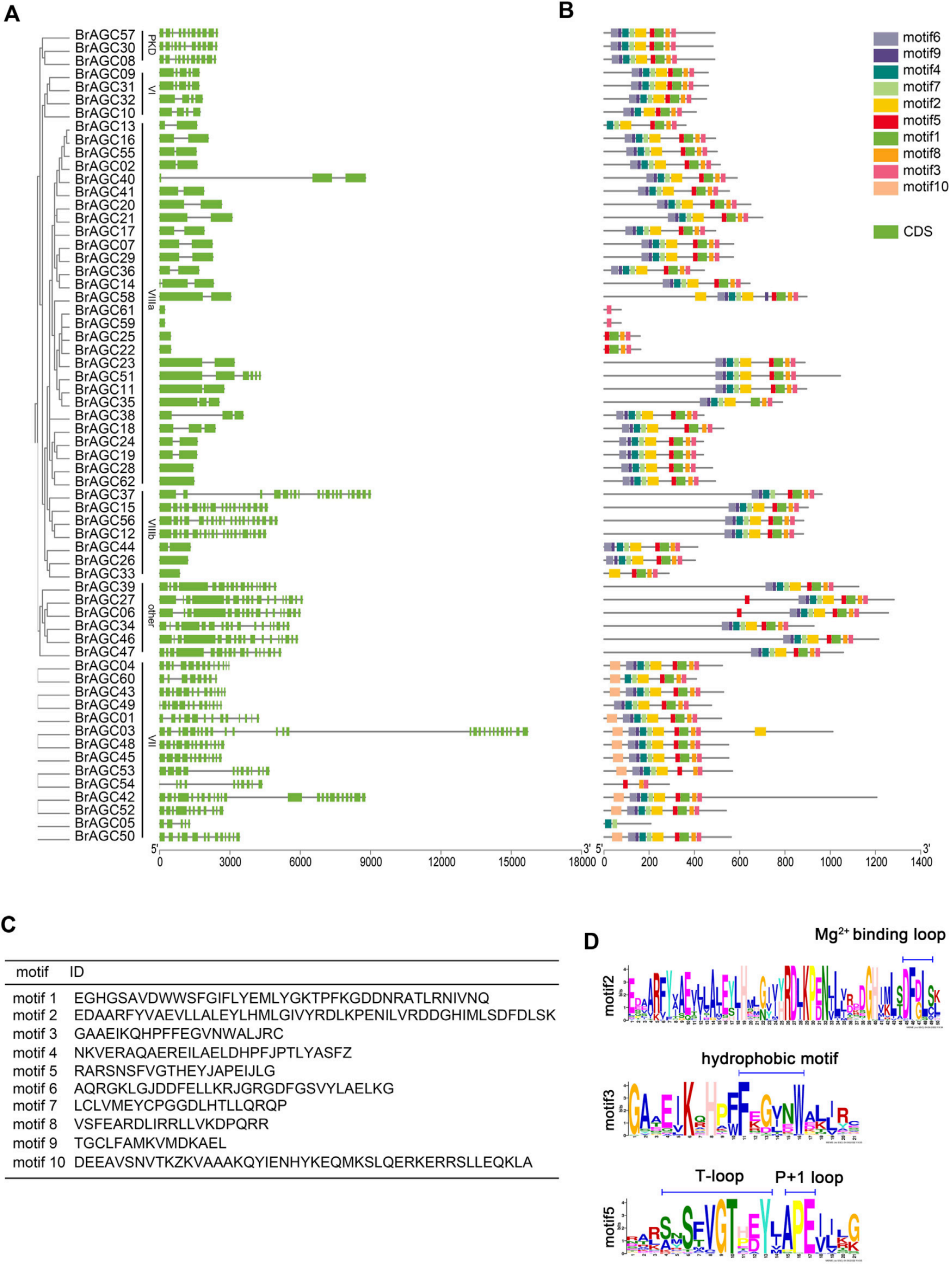
Gene structure and conserved motifs of *BrAGC* genes. **(A)** Composition and distribution of conserved motifs of *BrAGC* genes. **(B)** Exon–intron structure of *BrAGC* genes. Green box represents exons, and lines connecting exons are introns. **(C)** Ten conserved motif sequences of *BrAGC* genes. **(D)** Motif logo of motif2, motif3, and motif5.

To clarify the composition and function of *BrAGC* genes, we visualized and analyzed the exon–intron structure of *BrAGC* genes (Figure 2A). The length and distribution of exons and introns of *BrAGC* genes within subfamilies were similar, and the degree of similarity was positively correlated with clustering relationships. However, the structure of the coding regions of *BrAGC* genes varied among subfamilies. Among all *BrAGC* genes, 96% of genes in the AGCVIIIa subfamily and *BrAGC44*, *BrAGC26*, and *BrAGC33* in the AGCVIIIb subfamily lack or contain only 1 to 2 introns, whereas the remaining genes have more introns (e.g., up to 25 in *BrAGC03*).

We predicted 10 conserved motifs of *BrAGC* genes (Figures 2B,C). The number and distribution of motifs of different genes varied and ranged from 1 to 10. Motif6, 9, 4, 7, 2, 5, 1, 8, and 3 are highly conserved in *BrAGC* genes and exist in 77.4% of *BrAGC* genes in the order of 5′–3′. Motif2 contains the Mg^2+^-binding loop, and motif5 is located at its C-terminal and contains a T-loop and P+1 loop (Figure 2D), which constitutes the activation segment of the AGC protein kinase. These two motifs are highly conserved in *BrAGCs*, with 88.7% and 93.5% of the genes containing motif2 and motif5, respectively. In addition, motif3 contains the hydrophobic motif FxxxW, which is located at the C-terminus of the *BrAGC* genes (Figure 2D), and it is the most widely distributed motif. In AGCVII, 64.3% of the genes contain all 10 motifs, and motif10 only exists in this subfamily. We speculated that motif10 might contribute to the functional diversity of the AGCVII subfamily. The distribution of motifs of genes in the same subfamily gene was similar, and the degree of similarity was positively correlated with clustering relationships. These results are consistent with the findings of the gene structure analysis and confirm the subfamily division of *BrAGC* genes.

### Gene ontology analysis

**FIGURE 3:**
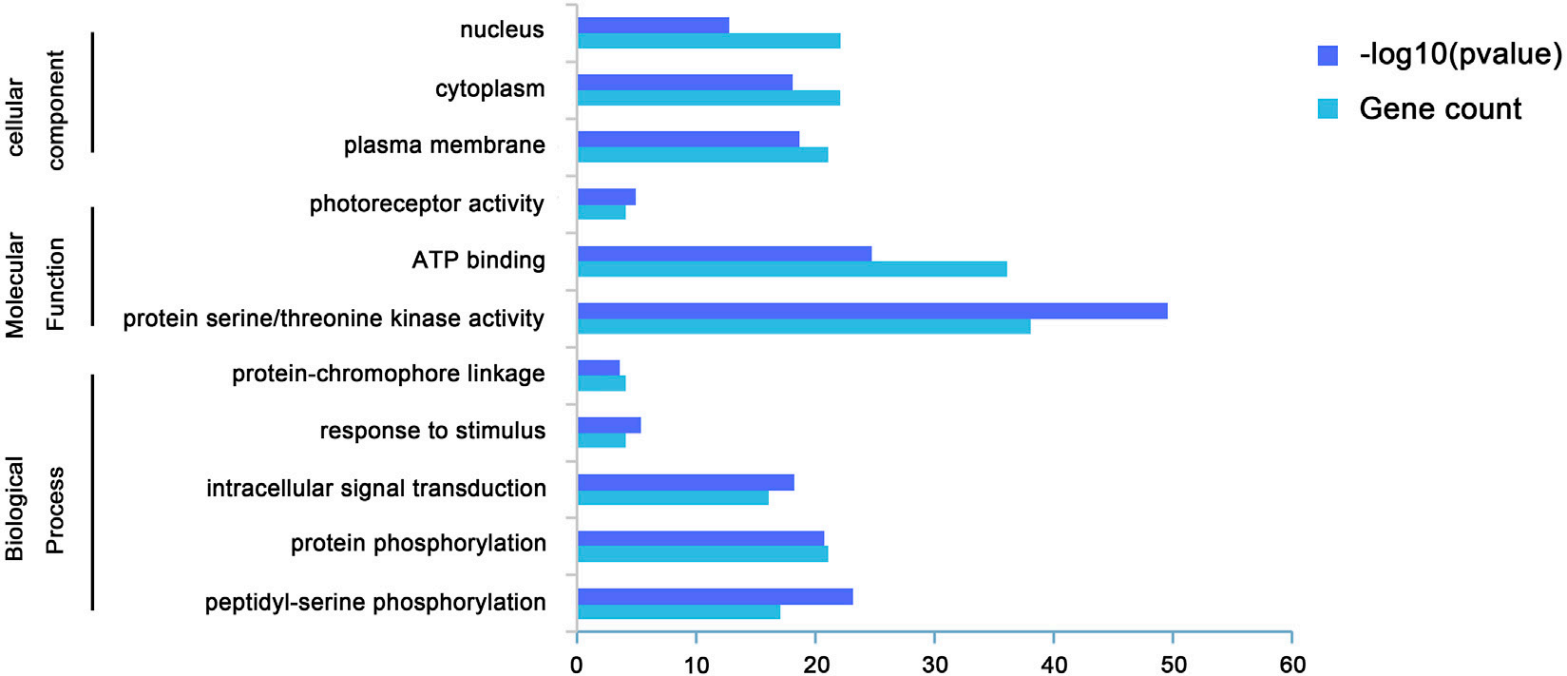
Gene Ontology of *BrAGC* genes in molecular function, biological process, and cellular component categories.

We performed GO analysis on *BrAGC* genes to determine their functions (Supplementary Table S4; Figure 3). GO is divided into three parts: biological process, molecular function, and cellular component. *BrAGC* genes is mainly involved in phosphorylation-related biological processes, with a total of 38 genes enriched in phosphorylation, followed by 16 genes enriched in intracellular signal transduction, 4 genes enriched in response to stimulus, and 4 genes enriched in protein-chromophore linkage. In the molecular function category, 38 genes were enriched in protein serine/threonine kinase activity, 36 genes were enriched in ATP binding, and 4 genes were enriched in photoreceptor activity.

### Collinearity analysis of *B. rapa AGC*

**FIGURE 4:**
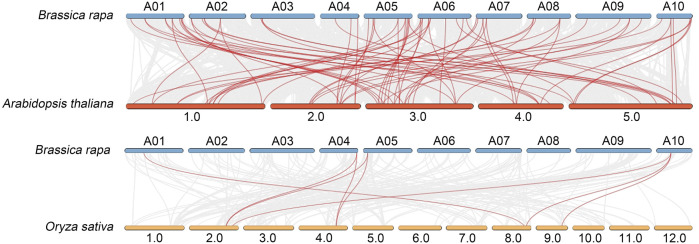
Collinearity analysis of *B. rapa*, *A. thaliana*, and *O. sativa AGC* genes. Homologous gene pairs of AGC protein kinases between species are linked by red lines.

To further clarify the origin and evolution of *BrAGC* genes, comparative syntenic relationships between *B. rapa* and two typical model plants, *A. thaliana* and *O. sativa*, were analyzed (Figure 4). There were 76 collinear pairs of *BrAGC* genes and *AtAGC* genes. There were only eight collinear pairs of *BrAGC* genes and *OsAGC* genes. These findings indicate that *B. rapa* and *A. thaliana* are closely related and functionally similar.

### *Cis*-elements in promoters

**FIGURE 5:**
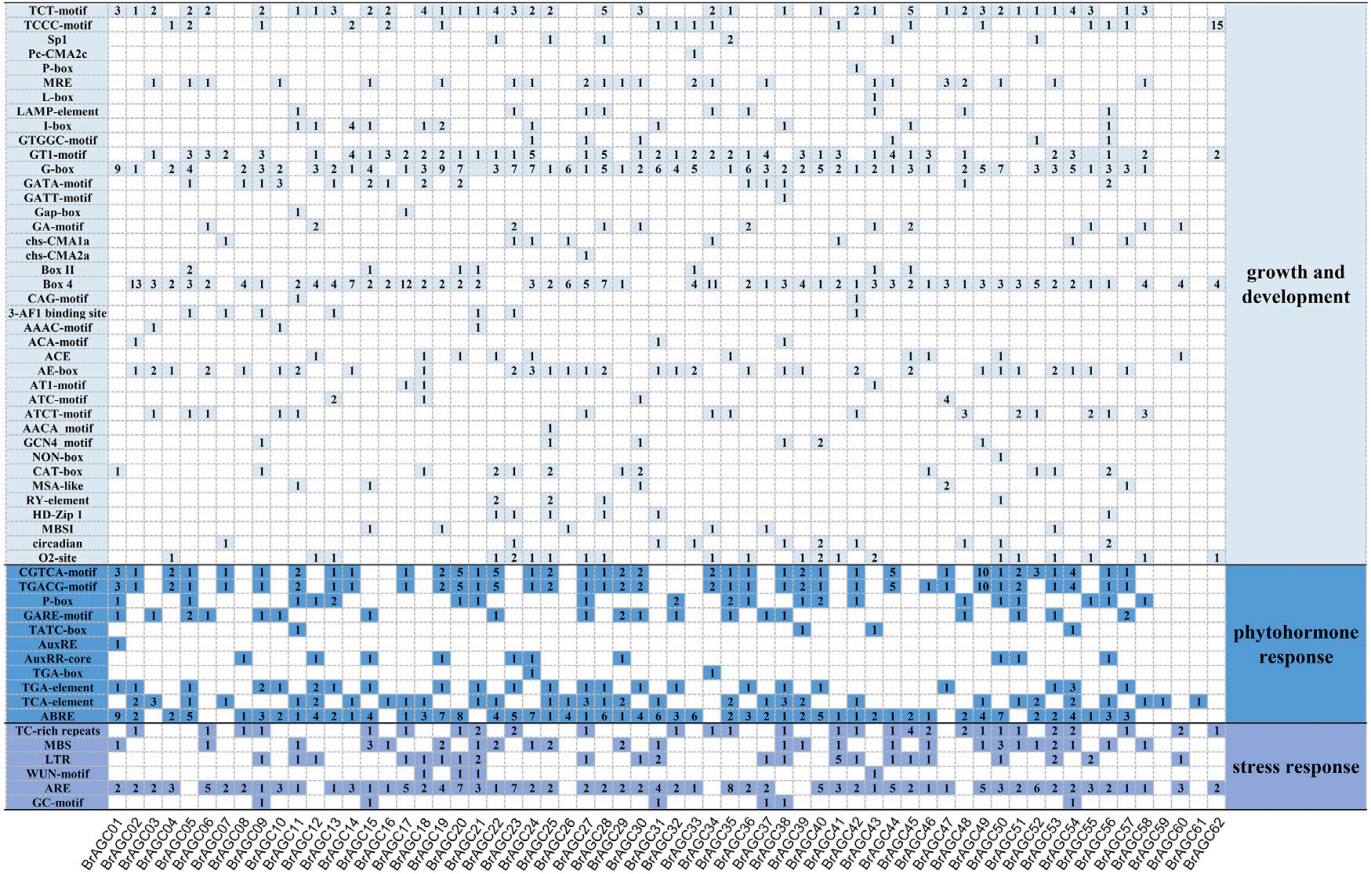
*Cis*-element analysis in the promoter region of *BrAGC* genes. The number of *cis*-elements in each gene is indicated by numbers.

The transcription of genes is mainly controlled by the recognition and binding of the DNA sequence motif of the *cis*-regulatory region by transcription factors (TFs), which activate or inhibit transcription to mediate the response to changes in the external environment (Qiu, 2003). To explore the mechanism by which *BrAGC* genes mediate the response to abiotic stress, the 2,000-bp upstream region of the coding sequences of *BrAGC* genes was used for *cis*-element prediction. A total of 1,532 *cis*-elements were predicted in the promoter region of *BrAGC* genes (Figure 5), and these were involved in growth and development response, phytohormone response, and stress response. There were 752 light-responsive elements in 62 *BrAGC* genes, and these were the most abundant *cis*-elements. In addition, the promoter region of *BrAGC* genes contains several phytohormone response elements; 79% of *BrAGC* genes contain abscisic acid-responsive elements (ABRE, total of 154); 60% of *BrAGC* genes contain MeJA-responsive elements (CGTCA-motif and TGACG-motif, total of 146); and 85% of *BrAGC* genes contain anaerobic induction elements (ARE, total of 141). The above data indicate that these elements might be involved in abiotic stress and the hormone response. Differences in the *cis*-elements of genes indicate that *BrAGC* genes might play different roles in plant growth and development.

### Expression patterns in different tissues

**FIGURE 6:**
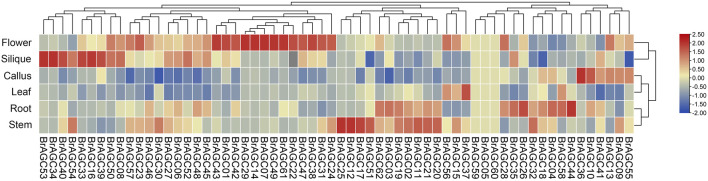
Expression profiles of *BrAGC* genes in different tissues of *B. rapa*. The abundance of each gene was measured using TPM.

To explore the potential functions of *BrAGC* genes, we analyzed the expression levels of *BrAGC* genes in the scallus, flower, leaf, root, silique, and stem of *B. rapa* (Figure 6; Supplementary Table S5). A total of 59 genes (except *BrAGC59*, *BrAGC05*, and *BrAGC60*) were expressed in at least one tissue, and 46 genes were expressed in all six tested tissues. Most *BrAGC* genes showed obvious expression differences among tissues, and genes preferentially expressed in flowers accounted for 35.5% (*N* = 22) of all genes; only 3.2% (*N* = 2) of genes were most highly expressed in the leaves. Some genes showed tissue-specific expression. For example, the expression level of *BrAGC12* in the stem was more than 10 times that in other tissues, and the expression level of *BrAGC33* in silique was nearly five times that in other tissues.

### Expression patterns during the self-incompatibility and cross-pollination response

**FIGURE 7:**
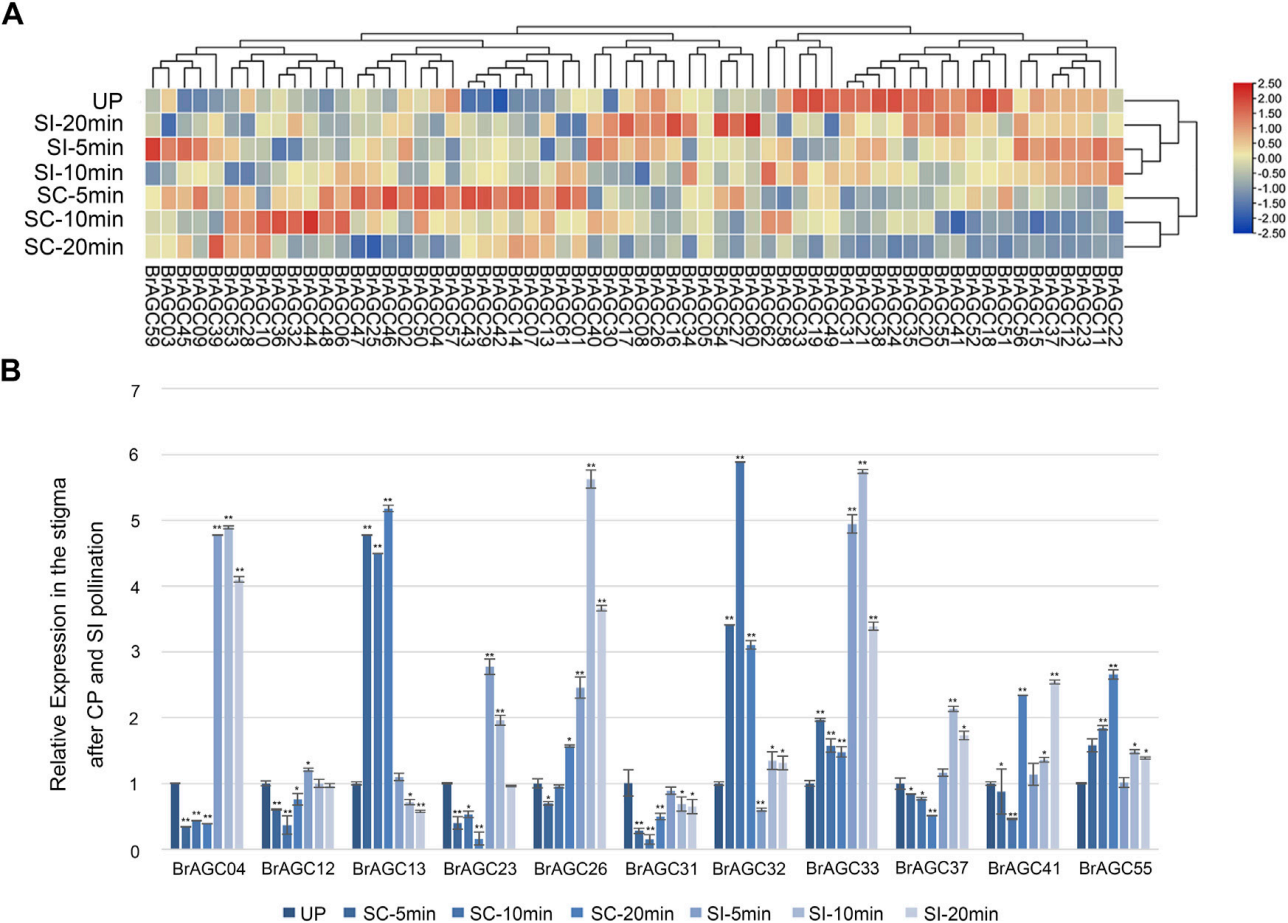
Expression profiles of *BrAGC* genes during the SI and CP response. **(A)** Heatmap showing the expression patterns of *BrAGC* genes in the UP stigma and under CP and SI pollination after 5, 10, and 20 min. **(B)** Transcriptional expression patterns of 11 *BrAGC* genes in the stigma after CP and SI pollination at 5, 10, and 20 min. The above experiments were performed using UP as the control; each group was subjected to three biological replicates, and error bars indicate standard errors. Asterisks above data bars indicate statistical significance (two-tailed *t*-test; *p < 0.05; **p < 0.01).

*B. rapa* is a typical self-incompatible vegetable. Some protein kinases have been shown to be involved in the regulation of SI and self-compatibility processes, such as FERONIA receptor kinase, which plays an important role in regulating the SI response (Li et al., 2016; Duan et al., 2020; Zhang et al., 2021). AGC protein kinases in *A. thaliana* have been shown to regulate the polar growth of pollen tubes (McCormick, 1993; Li et al., 2018). To study the function of *BrAGC* genes in the SI and CP response of *B. rapa*, the expression levels of *BrAGC* genes in unpollinated (UP) stigma and the stigma after CP and SI pollination at different times were detected by RNA-seq (Figure 7A; Supplementary Table S6), and 11 genes with high expression in the stigma were selected for qRT-PCR to verify the reliability of the RNA-seq results (Figure 7B). The relative expression levels of *BrAGC* genes in the stigma varied under different pollination conditions. After CP pollination, the relative expression levels of *BrAGC55*, *BrAGC13*, *BrAGC32*, and *BrAGC33* increased, and the most significant increases were observed for *BrAGC13* and *BrAGC32*; the expression levels of these genes were increased by more than five times compared with that of the control. In addition, the expression levels of *BrAGC23*, *BrAGC37*, *BrAGC12*, *BrAGC31*, and *BrAGC04* decreased. After SI pollination, the relative expression levels of *BrAGC23*, *BrAGC37*, *BrAGC26*, *BrAGC33*, *BrAGC32*, *BrAGC41*, and *BrAGC04* increased, and the most significant increases were observed for *BrAGC26*, *BrAGC33*, and *BrAGC04*; the expression levels of these genes were increased by more than five times compared with the expression of these genes in the control. The qRT-PCR results were consistent with the RNA-seq results; thus, the qRT-PCR results were used in subsequent analyses.

### Expression patterns in response to abiotic stress

Cold stress, salt stress, and drought stress are common threats in *B. rapa* production. To identify the key genes involved in responses to stress, 10 genes with high expression levels in *B. rapa* were selected for qRT-PCR detection after various stress treatments. *BrAGC* genes could respond to abiotic stress. Under the three stresses, the relative expression of *BrAGC37* and *BrAGC44* was significantly upregulated and downregulated, respectively. The expression patterns of the other genes varied under different types of stress.

**FIGURE 8:**
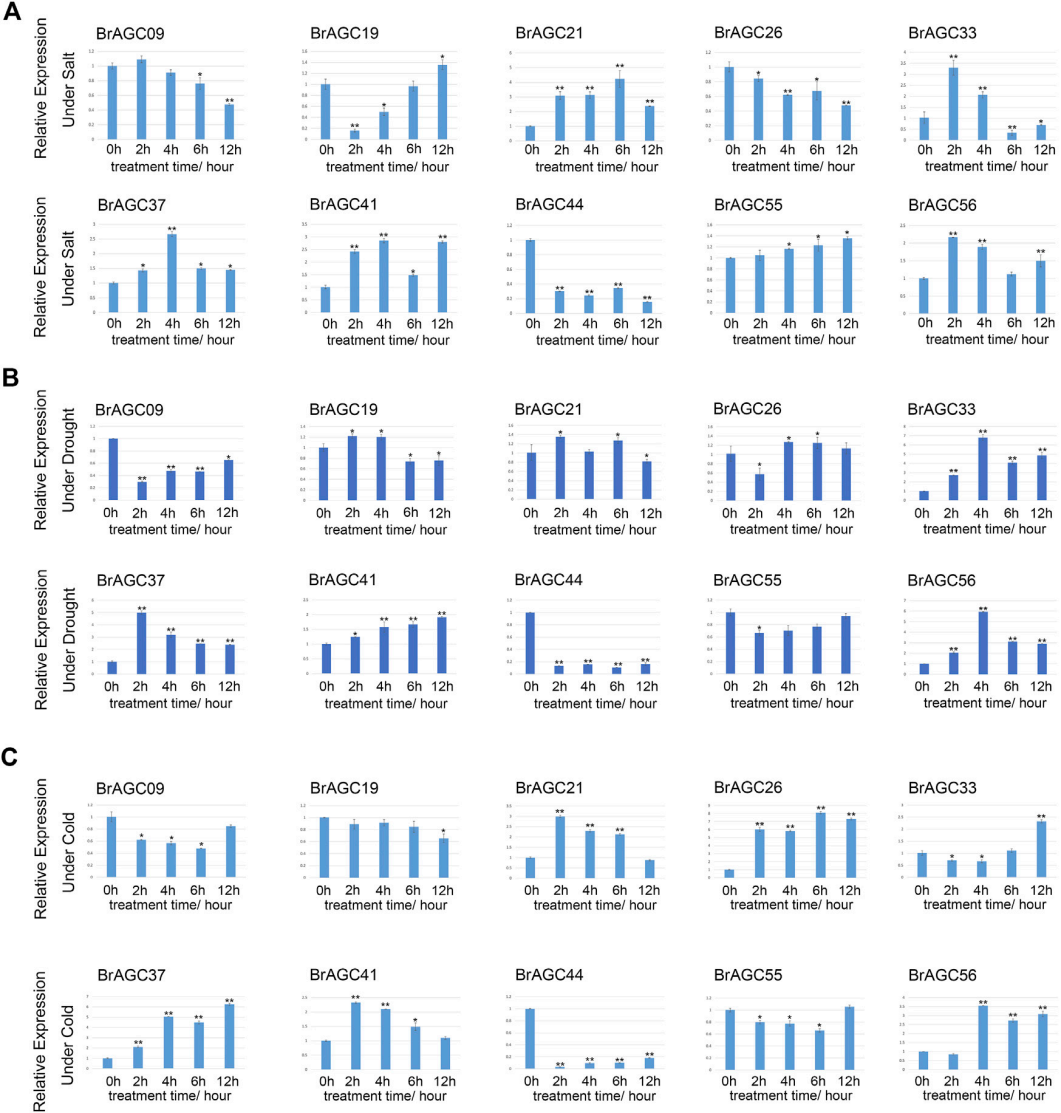
Expression profiles of 10 *BrAGC* genes under abiotic stress were analyzed by qRT-PCR. **(A)** Salt treatment. **(B)** Drought treatment. **(C)** Cold treatment. The above experiments were performed using 0 h as the control, and the treatment time was set to 2, 4, 6, and 12 h. Each group was subjected to three biological replicates, and error bars indicate standard errors. Asterisks above data bars indicate statistical significance (two-tailed *t*-test; *p < 0.05; **p < 0.01).

Under salt stress (Figure 8A), the relative expression levels of *BrAGC41*, *BrAGC37*, and *BrAGC21* were significantly upregulated at each time point after treatment. The expression of *BrAGC44* and *BrAGC26* was downregulated at each time point after treatment. The expression of *BrAGC33* and *BrAGC56* first increased and then decreased, and the expression of these genes peaked after 2 h of salt treatment.

Under drought stress (Figure 8B), the relative expression levels of *BrAGC37*, *BrAGC56*, and *BrAGC33* were significantly upregulated, and the expression levels peaked at 2 or 4 h. The expression levels of *BrAGC44*, *BrAGC55*, and *BrAGC09* were significantly downregulated, and the expression of *BrAGC44* showed the most significant downregulation among all genes.

The relative expression levels of *BrAGC21*, *BrAGC26*, *BrAGC37*, *BrAGC41*, and *BrAGC56* were higher under cold stress compared with under control conditions (Figure 8C). The relative expression levels of *BrAGC26* and *BrAGC37* were positively correlated with the treatment time. The relative expression levels of *BrAGC21* and *BrAGC41* peaked after 2 h of cold stress and then gradually decreased. The relative expression of *BrAGC44* decreased significantly after cold stress treatment.

### Protein-protein interaction networks

**FIGURE 9:**
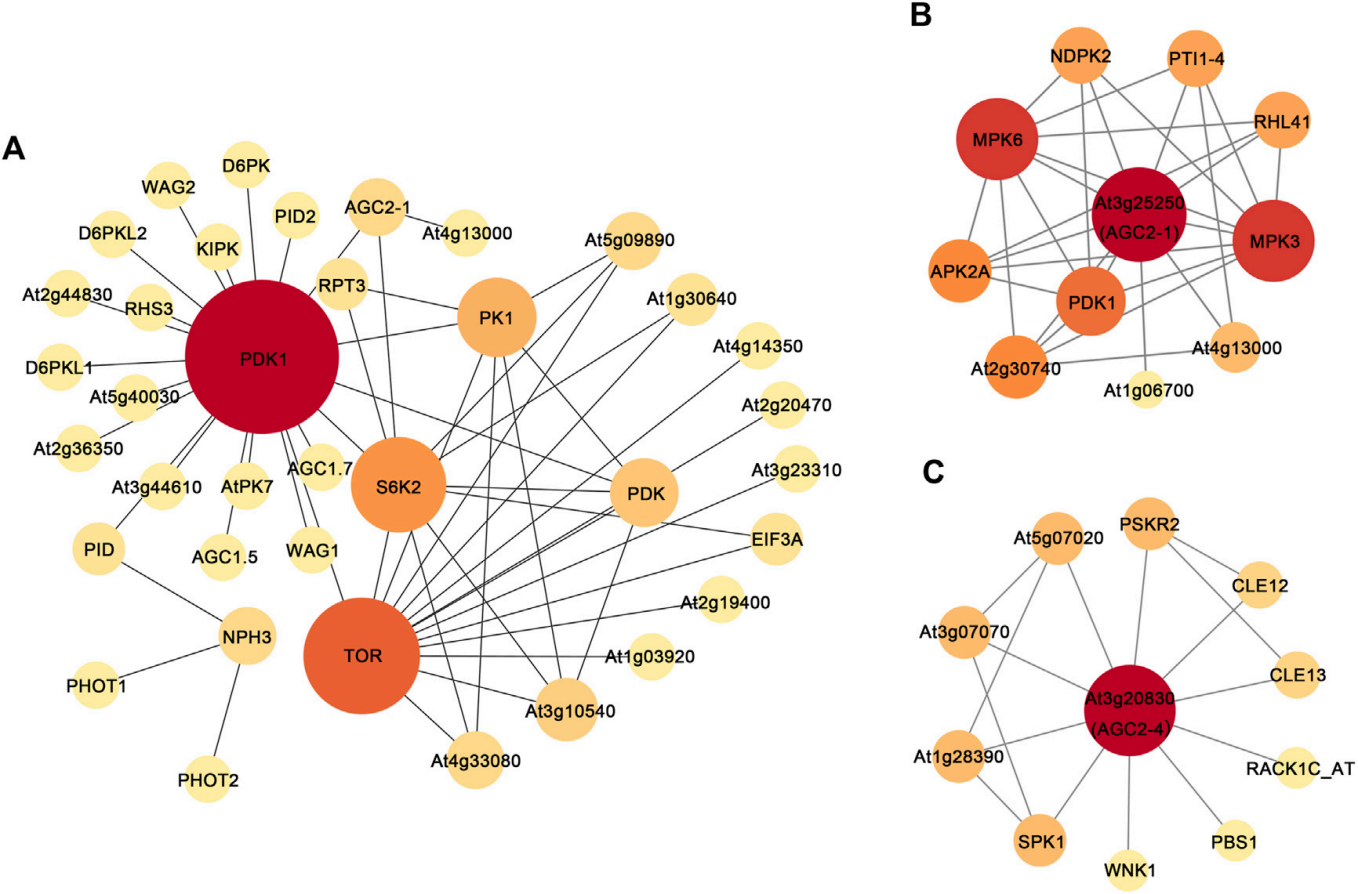
Protein-protein interaction networks of AGC protein kinase in *A. thaliana*. **(A)** All AtAGCs PPIs. **(B)** At3g25250 (*BrAGC44* homologous gene) PPIs. **(C)** At3g20830 (*BrAGC26* and *BrAGC33* homologous gene) PPIs. Each node represents a protein, and interacting protein connected by line. Node size and fill color shade are positively correlated with degree centrality.

AGC protein kinases are highly conserved in eukaryotes, and *BrAGC* genes are closely related to *AtAGC* genes. The functions of the *AtAGC* genes have been thoroughly studied. We performed PPI network analysis on *AtAGC* protein kinases to further determine their functions. A total of 44 nodes and 52 edges were predicted in the PPI networks (Figure 9A; Supplementary Table S7), and five functional partners of *AtAGC* genes, TOR, PDK, EIF3A, RPT3, and NPH3, were predicted. These genes play a role in stress responses (Ahn et al., 2011), auxin signal transduction (Deng et al., 2016; Tan et al., 2020), and plant phototropism response (Motchoulski and Liscum, 1999). This indicates that AGC protein kinases might regulate associated biological processes through its close relationship with these proteins. We also found that At3g25250 (*BrAGC44* homologous gene) interacted with zinc-finger (C2H2-type) protein (At5G59820, *AtZat12*) (Figure 9B). *AtZat12* has been shown to play a key role in ROS and abiotic stress signal transduction, and an interaction was detected between At3g20830 (*BrAGC26* and *BrAGC33* homologous gene) and At4g16340, a gene encoding guanine nucleotide exchange factor (GEF) (Figure 9C; Supplementary Table S7). These results further indicate that *BrAGC* genes have important and diverse function.

## Discussion

AGC protein kinases are a subgroup of serine/threonine protein kinases that are widespread in eukaryotes, and they play important roles in regulating plant growth and development, as well as resistance, immunity, and programmed cell death. No studies to date have identified the AGC protein kinases in *B. rapa*, and the functions of *BrAGC* genes have not yet been reported. We identified 62 *BrAGC* genes in *B. rapa* and analyzed their physicochemical properties, structural characteristics, and expression patterns. The results of this study will aid future studies of the functions of *BrAGC* genes.

Whole-genome duplication (WGD), segmental duplication, and tandem duplication are the main drivers of gene family expansion in species, and these processes can enrich the functional diversity of gene family members (Li et al., 2020). A total of 17 segmentally duplicated gene pairs were identified among *BrAGC* genes, and the number of segmentally duplicated gene pairs was 8.5 times that of the number of tandemly duplicated gene pairs. This indicates that segmental duplication is an important driver of the expansion of *BrAGC* genes in the *B. rapa* genome and that it has promoted the emergence of new functional genes in the *BrAGC* gene family (Cannon et al., 2004). The Ka/Ks ratio of duplicated gene pairs in *BrAGCs* was substantially less than 1, which suggests that *BrAGC* genes have experienced purifying selection. The number of *BrAGC* genes (*N* = 62) was higher than that of *AtAGC* genes (*N* = 39), which might reflect the three WGDs that *B. rapa* has experienced (common to all Brassicaceae members), as well as a *Brassica*-specific whole-genome triplication (WGT) event (Wang et al., 2011; Cheng et al., 2013). The diploid *B. rapa* genome has been rediploidized through chromosomal rearrangements and gene loss (Cai et al., 2021). Collinearity analysis between *BrAGCs* and *AGCs* in the model plants *A. thaliana* and *O. sativa* has shown that the number of collinear gene pairs of *BrAGCs* and *AtAGCs* was more than 9 times higher than that between *BrAGCs* and *OsAGCs*, indicating that the evolutionary relationships between *BrAGCs* and *AtAGCs* are closer than those between *BrAGCs* and *OsAGCs*. This allows us to speculate on the functions of *AtAGC* genes orthologous to *BrAGCs*. We also found that one *AtAGC* gene could be mapped to up to five *BrAGC* genes, which reflects the WGT event in *B. rapa*.

The activation segment of protein kinases usually includes the Mg^2+^-binding loop (DFG), T-loop, and the P+1 loop (Nolen et al., 2004; Taylor and Kornev, 2011), and the C-terminus of AGC protein kinases has a hydrophobic motif (FxxF) that regulates its interaction with PDK1, which is called the PDK1-interacting fragment (PIF). In plant AGC kinases, glycine (G) in the Mg^2+^-binding loop (DFG) is replaced by aspartic acid (D), and there are 36–118 aa insertion domains between the Mg^2+^-binding loop (DFDLS) and the T-loop (SFVGT), which are typical features of plant AGC kinases (Zegzouti et al., 2006). Analysis of the structural characteristics of *BrAGC* genes revealed that the characteristic motifs of plant AGC protein kinases are conserved in *BrAGCs*, such as motif3 containing the hydrophobic motif FxxxW, which is present in 98.4% of *BrAGC* genes. It can interact with the T-loop in motif5 to promote phosphorylation (Frödin et al., 2002). Special motifs also exist in different subfamilies. For example, motif10 exists at the N-terminus of 85.7% AGCVII subfamily genes, which is a motif specific to this subfamily. The conserved domain (NCBI Accession number: cl45907) corresponding to motif10 plays a role in the regulation of cellular functions such as cell morphogenesis, cell division, proliferation, and apoptosis (Lu et al., 2020), and the functions of the homologous genes of the subfamily in regulating cell division and cell morphology have been verified in yeast and other eukaryotes (Tamaskovic et al., 2003). Thus, the genes in the AGCVII subfamily might play a role in cellular metabolism.

Most plants in Brassicaceae have evolved SI to resist SI pollination and promote CP, which can help maintain the hybrid vigor and genetic diversity of plants (Takayama and Isogai, 2005). *BrAGCs* were preferentially expressed in flowers (Figure 5), and AGC protein kinases in *A. thaliana* have been shown to regulate the polar growth of pollen tubes (McCormick, 1993; Li et al., 2018). Suggesting that AGC protein kinases play an important role in sexual reproduction in plants. The expression levels of *BrAGC* genes in the stigma were detected at different times following SI and CP. The upregulated genes *BrAGC13* and *BrAGC32* after CP and the upregulated genes *BrAGC26*, *BrAGC33*, and *BrAGC04* after SI pollination might be involved in the interaction between pollen and the stigma in *B. rapa*. In addition, PPI network analysis showed that the homologous gene At3g20830 of *BrAGC26* and *BrAGC33* interacts with At4g16340. At4g16340 encodes SPIKE1 (SPK1), the guanine nucleotide exchange factor (GEF) in Arabidopsis. AGC1.5 in *A. thaliana* can directly interact with GEFs to control the polar growth of pollen tubes (Li et al., 2018). Therefore, *BrAGC26* and *BrAGC33* might play a role in regulating the pollen–pistil interaction; additional studies are needed to confirm this possibility.

*B. rapa* is often threatened by abiotic stress, and this has favored the evolution of mechanisms to resist abiotic stress (Nakashima and Yamaguchi-Shinozaki, 2013). More than 96% of AGCVIIIa genes and *BrAGC44*, *BrAGC26*, and *BrAGC33* in AGCVIIIb lack introns or only contain 1 to 2 introns; by contrast, other subfamilies have more introns. Previous studies have demonstrated that the expression levels of genes with fewer introns can rapidly change in response to stress (Jeffares et al., 2008). Fewer introns are conducive to the transcriptional regulation of genes under stress and facilitate stress responses. In other plants, AGCVIII kinase is involved in the regulation of plant growth and development and in responses to stress. We speculate that genes in the AGCVIII subfamily might play an important role in stress responses (Rentel et al., 2004; Devarenne et al., 2006; Petersen et al., 2009; Matsui et al., 2010; Zhu et al., 2015). Therefore, AGCVIII subfamily members might play important roles in the stress responses of *B. rapa*. To further predict the genes that might be involved in responses to abiotic stress, we analyzed the PPI networks of *AtAGC* genes and the *cis*-elements of *BrAGC* genes and characterized the expression patterns of some genes under low-temperature stress, salt stress, and drought stress. We found that TOR, a predicted functional partner of *AtAGC* genes, is closely related to several AtAGC protein kinases and controls plant growth under environmental stress (Ahn et al., 2011). AGC protein kinases can affect the kinase activity of TOR through phosphorylation (Hálová et al., 2013). Therefore, AGC protein kinases may regulate the response to external stress through cooperation with TOR. In addition, we found that the relative expression levels of *BrAGC21*, *BrAGC26*, *BrAGC37*, *BrAGC41*, and *BrAGC56* were upregulated under cold stress, and the promoter regions of *BrAGC21*, *BrAGC37*, and *BrAGC41* contained low temperature-responsive elements (LTRs). Under drought stress, the relative expression levels of *BrAGC33*, *BrAGC56*, and *BrAGC37* were upregulated, and *BrAGC56*, *BrAGC21*, and *BrAGC41* contained drought-inducible elements (MBS). Under salt stress, the relative expression levels of *BrAGC41*, *BrAGC37*, and *BrAGC21* were upregulated, and *BrAGC21* and *BrAGC41* contained defense and stress-responsive elements (TC-rich repeats). Phytohormone response elements are widely distributed in *BrAGC* genes, including those for gibberellin (P-box, GARE-motif, and TATC-box), auxin (AuxRE, AuxRR-core, TGA-box, and TGA-element), salicylic acid (TCA-element), and MeJA (CGTCA-motif and TGACG-motif), and AtAGC protein kinase can interact with PDK and TOR to regulate auxin signal transduction (Deng et al., 2016; Tan et al., 2020). Therefore, *BrAGC* genes may affect plant growth and development and environmental stress responses through the hormone signal regulation network. The promoter regions containing these elements have been shown to regulate the expression of related genes, which allows plants to respond to changes in the external environment. In addition, the expression of *BrAGC37* was upregulated under three types of abiotic stress, and the relative expression of *BrAGC44* was significantly decreased under these three abiotic stresses. Plants can enhance their resistance to other stresses after experiencing certain stresses; this might contribute to the cross-adaptation process of *B. rapa* and have a conserved function in the response to abiotic stress. ROS are important signaling molecules that play a role in the responses of plants to biotic and abiotic stresses, resistance to pathogen invasion, and the regulation of programmed cell death (Apel and Hirt, 2004). In many plants, the expression of AGC protein kinase member OXI is induced by oxidative stress; this protein plays key roles in plant defense and immunity. In addition, PPI network analysis predicted that *AtZat12* is a functional partner of *AtOxi1*. *AtZat12*; it was also predicted to be involved in ROS metabolism and play a role in various abiotic stresses (Rizhsky et al., 2004; Davletova et al., 2005). *BrAGC44* is orthologous to *AtOxi1*, and the relative expression of *BrAGC44* is significantly downregulated under the three abiotic stresses. Based on the above conclusions, *BrAGC44* might mediate the resistance of plants to stress by regulating ROS.

## Conclusion

We identified 62 *BrAGCs* in *B. rapa*. Based on the comprehensive analysis of sequence features, *cis*-elements, expression profiles of different tissues, abiotic stress tolerance, sexual reproductive processes, and the published data, we found that *BrAGC26*, *BrAGC33*, and *BrAGC44* likely regulate pollen–pistil interactions and abiotic stress tolerance, respectively.

## Data Availability

The data presented in the study are deposited in the NCBI GEO (http://www.ncbi.nlm.nih.gov/geo/) with the accession number GSE43245.
