# Supplementary material for: Genome-wide identification and expression analysis of BrAGC genes in Brassica rapa reveal their potential roles in sexual reproduction and abiotic stress tolerance
Source: Front Genet. 2022 Oct 26;13:1044853. doi: 10.3389/fgene.2022.1044853 (PMC9644056; doi:10.3389/fgene.2022.1044853)
Supplement: Supplementary file 4 [file DataSheet1.PDF]

Supplementary Table 1 Reference

Anthony R G, Henriques R, Helfer A, Mészáros T, Rios G, Testerink C, et al. (2004). A protein kinase target of a PDK1 signalling pathway is involved in root hair growth in Arabidopsis. Embo j, 23(3): 572-581. doi:10.1038/sj.emboj.7600068

Bai F, Watson J C, Walling J, Weeden N, Santner A A, Demason D A (2005). Molecular characterization and expression of PsPK2, a PINOID-like gene from pea (Pisum sativum). Plant Science, 168(5): 1281-1291. doi: 10.1016/j.plantsci.2005.01.005

Camehl I, Drzewiecki C, Vadassery J, Shahollari B, Sherameti I, Forzani C, et al. (2011). The OXI1 kinase pathway mediates Piriformospora indica-induced growth promotion in Arabidopsis. PLoS Pathog, 7(5): e1002051. doi:10.1371/journal.ppat.1002051

Dhonukshe P, Huang F, Galvan-Ampudia C S, Mähönen A P, Kleine-Vehn J, Xu J, et al. (2015). Plasma membrane-bound AGC3 kinases phosphorylate PIN auxin carriers at TPRXS(N/S) motifs to direct apical PIN recycling. Development, 142(13): 2386-2387. doi:10.1242/dev.127415

Ek-Ramos M J, Avila J, Cheng C, Martin G B, Devarenne T P (2010). The T-loop extension of the tomato protein kinase AvrPto-dependent Pto-interacting protein 3 (Adi3) directs nuclear localization for suppression of plant cell death. J Biol Chem, 285(23): 17584-17594. doi:10.1074/jbc.M110.117416

Elliott R C, Platten J D, Watson J C, Reid J B (2004). Phytochrome regulation of pea phototropin. J Plant Physiol, 161(3): 265-270. doi:10.1078/0176-1617-01228

Enugutti B, Kirchhelle C, Oelschner M, Torres Ruiz R A, Schliebner I, Leister D, et al. (2012). Regulation of planar growth by the Arabidopsis AGC protein kinase UNICORN. Proc Natl Acad Sci U S A, 109(37): 15060-15065. doi:10.1073/pnas.1205089109

Hammond R W, Zhao Y (2000). Characterization of a tomato protein kinase gene induced by infection by Potato spindle tuber viroid. Mol Plant Microbe Interact, 13(9): 903-910. doi:10.1094/mpmi.2000.13.9.903

He Y, Yan L, Ge C, Yao X F, Han X, Wang R, et al. (2019). PINOID Is Required for Formation of the Stigma and Style in Rice. Plant Physiol, 180(2): 926-936. doi:10.1104/pp.18.01389

Huang F, Kemel Zago M, Abas L, Van Marion A, Galván-Ampudia C S, Offringa R (2010). Phosphorylation of conserved PIN motifs directs Arabidopsis PIN1 polarity and auxin transport. The Plant Cell, 22(4): 1129-1142. doi:10.1105/tpc.109.072678

Kanegae H, Tahir M, Savazzini F, Yamamoto K, Yano M, Sasaki T, et al. (2000). Rice NPH1 homologues, OsNPH1a and OsNPH1b, are differently photoregulated. Plant Cell Physiol, 41(4): 415-423. doi:10.1093/pcp/41.4.415

Khanna R, Lin X, Watson J C (1999). Photoregulated expression of the PsPK3 and PsPK5 genes in pea seedlings. Plant Mol Biol, 39(2): 231-242. doi:10.1023/a:1006154203639

Lin X, Feng X H, Watson J C (1991). Differential accumulation of transcripts encoding protein kinase homologs in greening pea seedlings. Proc Natl Acad Sci U S A,

88(16): 6951-6955. doi:10.1073/pnas.88.16.6951
Matsui H, Miyao A, Takahashi A, Hirochika H (2010). Pdk1 kinase regulates basal disease resistance through the OsOxi1-OsPti1a phosphorylation cascade in rice. Plant Cell Physiol, 51(12): 2082-2091. doi:10.1093/pcp/pcq167
Mcsteen P, Malcomber S, Skirpan A, Lunde C, Wu X, Kellogg E, et al. (2007). barren inflorescence2 Encodes a co-ortholog of the PINOID serine/threonine kinase and is required for organogenesis during inflorescence and vegetative development in maize. Plant Physiol, 144(2): 1000-1011. doi:10.1104/pp.107.098558
Michniewicz M, Zago M K, Abas L, Weijers D, Schweighofer A, Meskiene I, et al. (2007). Antagonistic regulation of PIN phosphorylation by PP2A and PINOID directs auxin flux. Cell, 130(6): 1044-1056. doi:10.1016/j.cell.2007.07.033
Morita Y, Kyozuka J (2007). Characterization of OsPID, the rice ortholog of PINOID, and its possible involvement in the control of polar auxin transport. Plant Cell Physiol, 48(3): 540-549. doi:10.1093/pcp/pcm024
Pislariu C I, Dickstein R (2007). An IRE-like AGC kinase gene, MtIRE, has unique expression in the invasion zone of developing root nodules in Medicago truncatula. Plant Physiol, 144(2): 682-694. doi:10.1104/pp.106.092494
Santner A A, Watson J C (2006). The WAG1 and WAG2 protein kinases negatively regulate root waving in Arabidopsis. Plant J, 45(5): 752-764. doi:10.1111/j.1365-313X.2005.02641.x
Turck F, Zilbermann F, Kozma S C, Thomas G, Nagy F (2004). Phytohormones participate in an S6 kinase signal transduction pathway in Arabidopsis. Plant Physiol, 134(4): 1527-1535. doi:10.1104/pp.103.035873
Xu M, Tang D, Cheng X, Zhang J, Tang Y, Tao Q, et al. (2019). OsPINOID Regulates Stigma and Ovule Initiation through Maintenance of the Floral Meristem by Auxin Signaling. Plant Physiol, 180(2): 952-965. doi:10.1104/pp.18.01385
Zourelidou M, Müller I, Willige B C, Nill C, Jikumaru Y, Li H, et al. (2009). The polarly localized D6 PROTEIN KINASE is required for efficient auxin transport in Arabidopsis thaliana. Development, 136(4): 627-636. doi:10.1242/dev.028365
